# Hypercalcemia of malignancy caused by parathyroid hormone-related peptide-secreting pancreatic neuroendocrine tumors (PTHrP-PNETs): Case Report

**DOI:** 10.3389/fonc.2023.1197288

**Published:** 2023-06-12

**Authors:** Stephanie Pitts, Amit Mahipal, David Bajor, Amr Mohamed

**Affiliations:** ^1^ Department of Medicine, University Hospitals (UH) Cleveland Medical Center, Cleveland, OH, United States; ^2^ Division of Hematology and Medical Oncology, University Hospitals (UH) Seidman Cancer Center Case Comprehensive Cancer Center, Case Western Reserve University, Cleveland, OH, United States

**Keywords:** management, hypercalcemia, PTHrP, pancreatic, neuroendocrine tumors

## Abstract

Parathyroid hormone-related protein (PTHrP) secretion is occasionally detected in various solid tumors such as renal cell carcinoma and lung cancers. It is considered quite rare for neuroendocrine tumors with only few published case reports. We reviewed the current literature and summarized a case report of a patient with metastatic pancreatic neuroendocrine tumor (PNET) presenting with hypercalcemia due to elevation of PTHrP. The patient had histological confirmation of well-differentiated PNET and developed hypercalcemia years after his initial diagnosis. In our case report, evaluation showed intact parathyroid hormone (PTH) in the setting of concomitant elevation of PTHrP. The patient’s hypercalcemia and PTHrP levels were improved by using a long-acting somatostatin analogue. In addition, we reviewed the current literature regarding the optimal management of malignant hypercalcemia due to PTHrP-producing PNETs.

## Introduction

Pancreatic neuroendocrine tumors (PNETs), also known as islet cell tumors, are a rare type of neoplasm arising from the endocrine tissue of the pancreas (1). There are many classifications for PNETs according to histological differentiation, grade, stage, tumor burden, somatostatin expression, and functionality. Functionality is based on secretion of a variety of peptide hormones such as insulin, glucagon, vasoactive intestinal peptide (VIP), gastrin and somatostatin. PNETs are considered functional tumors if they are secreting one of these hormones and patients have associated clinical symptoms. PNETs are considered non-functional if they are not secreting hormones (2). Most PNETs are found to be non-functioning tumors (50-75%). Previous reports have demonstrated that PNETs are capable of secreting parathyroid hormone-related peptide (PTHrP) causing hypercalcemia of malignancy (3, 4).

PTHrP was discovered in 1987 and is the hormone responsible for hypercalcemia of malignancy in many solid tumors such as renal cell carcinoma, lung, breast, and head and neck cancers (3). Here, we present a case and corresponding review of the literature in regards to PTHrP-secreting PNETs. We discuss clinical presentation, challenges related to management of PTHrP-induced hypercalcemia, disease progression, and prognosis.

## Case report

**Figure 1:**
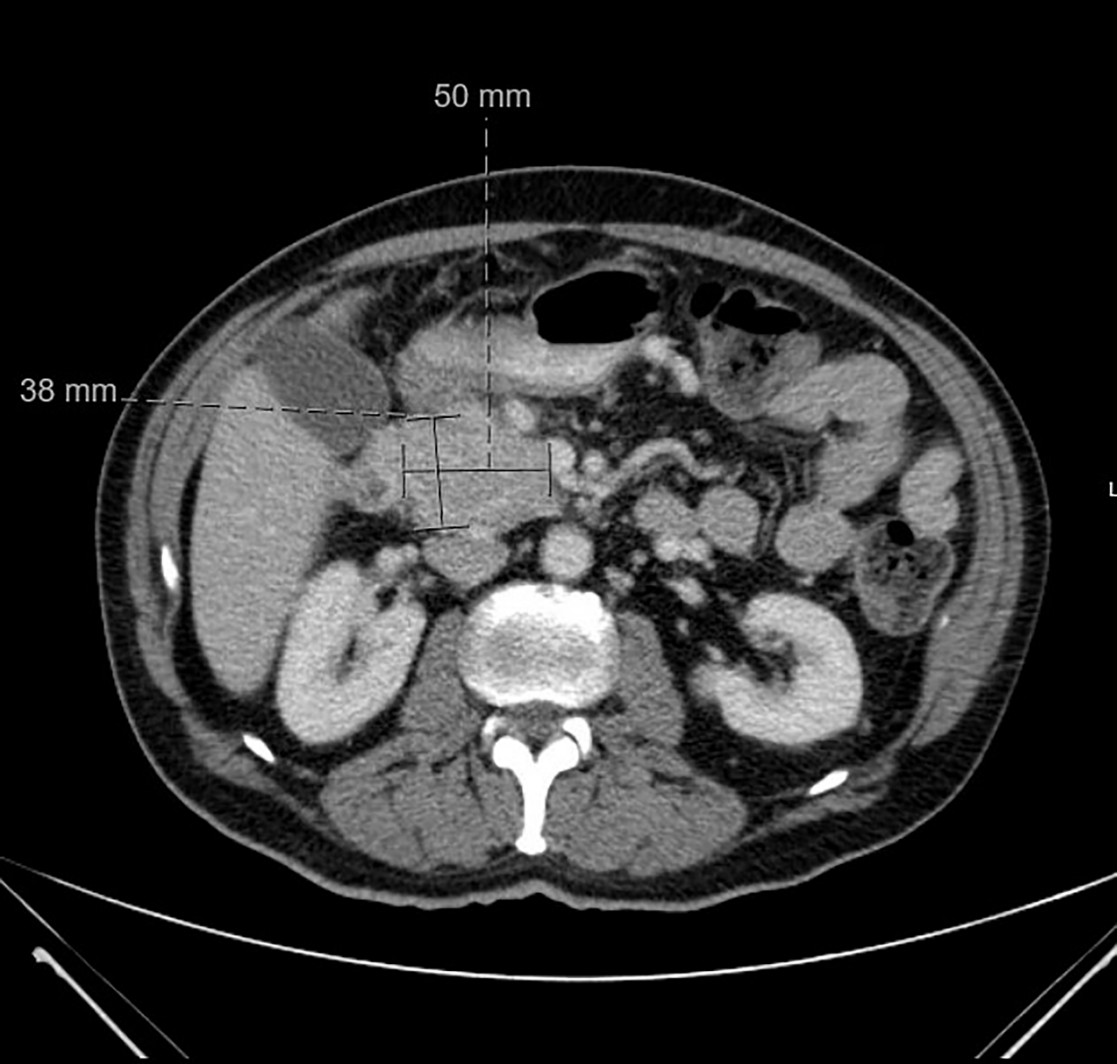
CT abdomen and pelvis at time of diagnosis. CT abdomen and pelvis with IV contrast at time of diagnosis was positive for pancreatic head neoplasm measured at 5 x 3.8 cm.

A 64 year old male with a remote history of prostate cancer and type 2 diabetes mellitus presented to the emergency department with syncope and 10 lb weight loss over 6 months. A computed tomography (CT) of his abdomen and pelvis was performed which showed a pancreatic head mass with no associated liver lesions or enlarged lymph nodes (**Figure 1**). An MRI of his liver further delineated a 5.3cm pancreatic head mass. Endoscopic ultrasound (EUS) with fine needle aspiration (FNA) of the mass revealed an initial diagnosis of pancreatic adenocarcinoma. Further staging showed that the cancer was only locally advanced. The patient was treated as borderline pancreatic cancer and he underwent neoadjuvant FOLFIRINOX (folinic acid, fluorouracil, irinotecan, and oxaliplatin) and then chemoradiation with gemcitabine followed by a pancreaticoduodenectomy (whipple). Final surgical pathology obtained from the Whipple revealed pancreatic neuroendocrine tumor (Grade 1, Ki-67 2.5%). By immunohistochemistry, the tumor cells were positive for synaptophysin and chromogranin and negative for PAX-8.

**Figure 2:**
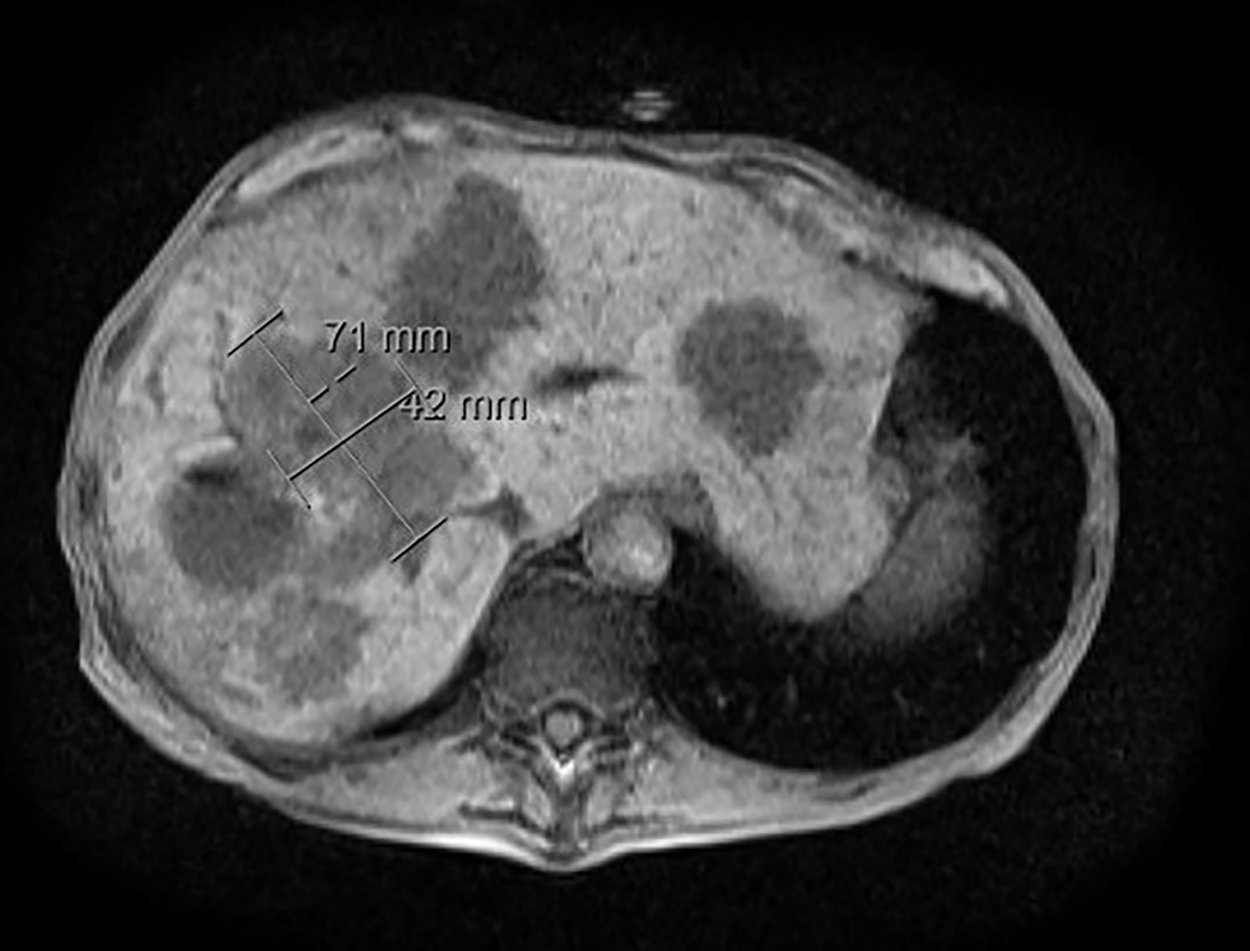
MRI abdomen at time of progression. MRI abdomen with/without contrast at time of progression showed metastatic hypervascular hepatic masses.

The patient subsequently did well for approximately two years until a surveillance CT abdomen and pelvis revealed multiple new liver lesions which have been confirmed with MRI of the abdomen consistent with new metastatic disease (**Figure 2**). He started Sandostatin LAR (octreotide) intramuscular (IM) injections 30 mg every 28 days. Approximately four months later, follow-up CT abdomen and pelvis showed further progression of the liver lesions with no evidence of extrahepatic lesions and the patient underwent bland embolization. Restaging CT abdomen and pelvis scans showed stability of his metastatic liver lesions. During this time period, however, the patient developed recurrent liver abscesses requiring multiple courses of various antibiotics and intermittent stent placement. PET-Gallium 68 (PET-Ga68) showed disease progression in the liver, he was started on Sunitinib and was stable for almost two years. Afterwards, restaging scans showed disease progression in the liver and extrahepatic lymph nodes, which were all somatostatin avid on the PET-Ga68. At this point, patient was referred to our institution for a second opinion and he was started peptide receptor radionuclide therapy (PRRT) and completed four cycles. During the follow up period after PRRT, the patient was noted to have hypercalcemia (between 11-12 mg/dL). He only reported chronic symptoms of nausea, weight loss, and fatigue. Parathyroid hormone (PTH) was suppressed to 7.7 pg/mL and PTHrP was found to be elevated at 75 pmol/L. He was treated with intravenous (i.v.) hydration, calcitonin, and i.v pamidronate during multiple hospital admissions due to hypercalcemia. Afterwards he was restarted on Sandostatin LAR (long-acting octreotide) 30mg IM injection every 28 days due to persistently elevated PTHrP levels and hypercalcemia; this resulted in stabilization of his calcium level in the normal range. However, the patient continued to decline due to his multiple comorbidities including recurrent liver abscesses. Due to poor performance status, anorexia and severe fatigue, the patient declined further treatments and opted for hospice care.

## Discussion

PNETs are a group of heterogeneous tumors that have various classifications according to histological differentiation, grade, stage, tumor burden, and functionality. According to functionality, PNETs can be divided into functional and non-functional tumors. A majority of PNETs are considered non-functional (55-60%). PNETs that are functioning (hormone-secreting) are classified according to the predominant hormone they secrete and the corresponding clinical syndrome they cause such as insulinoma, gastrinoma, VIPoma, glucagonoma, and somatostatinoma. Rarely PNETs may produce other hormones such as PTHrP. Humoral hypercalcemia of malignancy, mediated by PTHrP in PNETs, remains quite rare and is usually associated with poor prognosis (5). PTHrP-secreting PNETs have only been described previously in case reports. The first described cases of hypercalcemia related to PTHrP in the setting of PNET was in 1990 (3). In the following years, some case reports and case-series were published describing cases of hypercalcemia related to PTHrP in the setting of PNETs (3, 4).

PTHrP is a polypeptide that is expressed in a wide variety of neuroendocrine cells and has revealed some homology with PTH, allowing it to act at the PTH-1 receptor site and simulate similar actions, including increases in bone resorption and distal tubular calcium reabsorption (6). PTHrP-induced hypercalcemia of malignancy is more commonly associated with malignant tumors of lung, breast, kidney, and head and neck and less frequently with PNETs (5). Secretion of multiple hormones by PNETs is uncommon, however the most commonly reported hormones secreted along with PTHrP include gastrin, VIP and somatostatin (6–12). Our patient only had a PTHrP-secreting tumor with no other detected hormonal secretion.

In terms of demographics for PTHrP-secreting PNETs, there is an equal distribution between men and women, which is consistent with the general epidemiology of PNETs. Age varied widely, though the majority of patients presented in the fourth to sixth decade of life, which is again consistent with the general presentation of PNETs (7, 13).

For most of the patients in the published case series, the development of symptomatic hypercalcemia occurred between months and several years after the diagnosis of PNETs, however for a rare few, hypercalcemia was present at presentation (**Table 1**) (11, 14). Our patient was diagnosed with hypercalcemia two years after his initial diagnosis with PNET. One factor that appeared to be important in the development of hypercalcemia was the presence of metastases. The vast majority of patients with hypercalcemia of malignancy and PNETs had metastatic disease with the most commonly involved site being the liver. In our case report, the patient had synchronous liver metastasis at the time of his presentation with PTHrP-related hypercalcemia.

**Table 1:** Previous case reports with PTHrP-hypersecreting NETs.

Case Number	Age (Year)	Sex	Primary tumor	Calcium (g/dl)	PTHrP (pmol/L)	Tumor grade/Ki67%	Reference Number
1	49	F	PNET	13.6	146	G1/<2%	(11)
2	53	M	PNET	14.7	138	G2/5-10%	
3	52	M	PNET	14.8	16	G2/<5%	(11)
4	40	F	UNK	11	9.1	G2/10%	(11)
5	54	M	PNET	17.4	912	G1/2%	(11)
6	41.1	M	PNET	4.81	2.6–6.0	UNK	(15)
7	58.3	M	PNET	5.86	2.9 –5.2	G1	(15)
8	40	F	PNET	5.36	2.3	UNK	(15)
9	52.9	M	PNET	5.39	2.0 –2.6	G3	(15)
10	61.1	M	PNET	4.65	5.4–16.3	G1	(15)
11	60.8	M	UNK	5.51	2.5	G2	(15)
12	38.3	M	PNET	4.30	2.5	G1	(15)
13	42.2	F	PNET	5.68	3.1	UNK	(15)
14	51.3	F	PNET	4.72	3.4	UNK	(15)
15	49.5	F	PNET	5.75	1.8	G1	(15)

M, Male; F, Female; PNET, Pancreatic Neuroendocrine Tumors; G1, Grade 1; G2, Grade 2; G3, Grade 3; UNK, Unkown.

Regarding clinical signs and symptoms, a majority of the patients were symptomatic with hypercalcemia regardless of whether they had already been diagnosed with PNETs. The most commonly reported symptoms included abdominal pain, poor appetite, nausea, vomiting, constipation, confusion, and weight loss, which are typical symptoms related to hypercalcemia. For precise diagnosis in these cases, it is important to check both a serum PTH and PTHrP in patients with PNETs who present with hypercalcemia. Our patient had elevated PTHrP with concomitant suppressed PTH which confirms that the secretion of endogenous PTH was suppressed by PTHrP.

In terms of treatment, the hypercalcemia due to PTHrP in PNETs can be challenging to control. The treatment should concentrate on two aspects including acute treatment of hypercalcemia and reduction of PTHrP by cytoreduction of the tumors. The treatment of hypercalcemia related to PTHrP in PNETs requires standard management including hydration, forced diuresis, calcitonin, and bisphosphonate therapies. However, in order to ultimately prevent recurrent hypercalcemia, definitive treatment of the PNETs is necessary. Ultimately, if possible, the most effective way to achieve remission from PTHrP-induced hypercalcemia caused by PNETs includes surgical debulking though many patients may not be eligible. An alternative method of cytoreduction which can be considered is liver directed therapy (radioembolization, bland embolization, or chemoembolization) in patients who have high liver tumor burden. Previous case series documented partial control of disease progression and recurrence of hypercalcemia through arterial chemoembolization (**Table 2**) (11, 14–16). Regarding medical management, somatostatin analogs (SSAs) are used to control hormonal secretion in functional NETs. Given that approximately 80% of gastroenteropancreatic neuroendocrine tumors (GEP-NETs) express somatostatin receptors, especially subtype 2 (SSTR2), SSAs have been used to control endocrine symptoms related to hormonal secretion and tumor growth (17) In randomized phase III trials, SSAs were associated with symptom control in up to 70% of patients and biochemical response in 30-40% (18, 19). Published case reports have shown that SSAs resulted in normalization of serum calcium levels in patients with PTHrP-induced hypercalcemia (14). However, other treatments have evolved, including peptide receptor radionuclide therapy (PRRT) which can be used in order to control hormonal secretion (20), there is insufficient data to confirm their benefits in PTHrP induced hypercalcemia. There are only some case reports for PRRT therapy resulted in significantly decreased serum calcium levels and plasma PTHrP levels, with long-term responses in some case reports reaching up to 40 months (21). Another option is cytotoxic chemotherapy with temozolomide and capecitabine (TC) which may help in reducing tumor burden and therefore controlling hypercalcemia especially in patients with PNETs. Based on retrospective data and a cooperative group trial (ECOG 2211), TC regimen was associated with a response rate of 39.7% and improvement in both progression-free survival (PFS) and overall survival (OS) (22). TC activity was mainly studied in PNETs and their activity is inferior in non-pancreatic NETs.

**Table 2:** Therapeutic interventions for PTHrP-hypersecreting NET reported cases.

Treatment	Number of patients	Reference Number
**Total number of patients**	**15**	(11, 15)
IV normal saline	10	(11, 15)
Bisphosphonate Derivative	8	(11, 15)
Somatostatin analogues	9	(11, 15)
Liver directed therapies (TACE or Y90)	6	(11, 15)
Surgery	2	(11, 15)
Chemotherapy	4	(11, 15)
Targeted therapies (Sunitinib)	2	(11, 15)
Peptide Receptor Radionuclide Therapy (PRRT)	5	(11, 15)

TACE, Transarterial chemoembolization; Y90, Yttrium90- Radioembolization.

Reduction in levels of hypercalcemia and PTHrP can be considered a good tumor marker to assess response to medical treatments, and refractory or worsening of hypercalcemia generally reflects disease progression (5). In regards to the prognostic value of PTHrP-secreting NETs, hypercalcemia due to PTHrP seems to have a negative impact on overall survival (23). Previous data reported that the overall survival in the patients with PTHrP-secreting NETs was significantly shorter compared to those with normocalcemia (14, 23). Therefore, early diagnosis and management of PTHrP induced hypercalcemia will significantly impact outcome in patients with metastatic GEP-NETs.

## In conclusion

Hypercalcemia is an uncommon presentation of functional NETs and is often mediated by PTHrP. It seems to impact survival and is considered a poor prognostic marker. Early diagnosis and management is essential for these patients to improve outcomes. With recent advances in medical management of metastatic GEP-NETs, it is important to discuss them in a multidisciplinary fashion to personalize these treatments for tumor burden and hormonal secretion control.

## Data availability statement

The original contributions presented in the study are included in the article/supplementary material. Further inquiries can be directed to the corresponding author.

## Ethics statement

The study was conducted in accordance with the Declaration of Helsinki (as revised in 2013). The study was approved by institutional ethics committee. (UH IRB,STUDY20191593). Written informed consent was obtained for the publication of this case report.

## Author contributions

SP: writing the manuscript. AMo: writing and editing the manuscript. All authors contributed to the article and approved the submitted version.
